# Computer-assisted sperm analysis of the epididymal spermatozoa in dromedary camels suffering from penile and preputial pathological problems

**DOI:** 10.3389/fvets.2025.1537708

**Published:** 2025-03-14

**Authors:** Montaser Elsayed Ali, Min Gao, Walaa M. Essawi, Ahmed Yassen M. Osman, Mohamed K. Hussein, Mohamed Abdelrahman, Fatimah A. Al-Saeed, Hassan A. Hussein, Yong-bin Liu, Ragab Hassan Mohamed

**Affiliations:** ^1^Department of Animal Productions, Faculty of Agriculture, Al-Azhar University, Assiut, Egypt; ^2^State Key Laboratory of Reproductive Regulation & Breeding of Grassland Livestock, Inner Mongolia University, Hohhot, China; ^3^Department of Theriogenology, Faculty of Veterinary Medicine, Aswan University, Aswan, Egypt; ^4^Department of Food Hygiene, Faculty of Veterinary Medicine, Aswan University, Aswan, Egypt; ^5^Department of Animal Production, Faculty of Agriculture, Assuit University, Asyut, Egypt; ^6^Department of Biology, College of Science, King Khalid University, Abha, Saudi Arabia; ^7^Department of Theriogenology, Faculty of Veterinary Medicine, Assiut University, Assiut, Egypt; ^8^Faculty of Veterinary Medicine, Sphinx University, Assiut, Egypt; ^9^Department of Animal Genetics, Breeding, and Reproduction, College of Animal Science, Inner Mongolia Agricultural University, Hohhot, China

**Keywords:** balanoposthitis, penile trauma, preputial prolapse, phimosis, penile tumors

## Abstract

Dromedary camels are the most vulnerable animals to penile and preputial pathology due to the aggressive nature of mating and injuries during transit, which could impair the semen quality. Hence, this study was performed to monitor the penile and preputial pathological conditions in male dromedary camels, as well as their impact on epididymal spermatozoa, by utilizing computer-assisted sperm analysis (CASA) and correlation analysis with testosterone concentrations. A total of 60 male dromedary camels were examined for penile and preputial pathological problems. The camels were grouped based on the absence or presence of the pathology conditions: (i) camels with normal penis and prepuce, (ii) camels with balanoposthitis, (iii) camels with penile trauma, (iv) camels with prolapsed prepuce, (v) camels with phimosis, and (vi) camels with penile tumors. The results revealed that there were significant increases in sperm concentration (Conc., M/ml), curvilinear velocity (VCL, *μ*m/s), eat-cross frequency (BCF, Hz), and straightness (STR, VSL/VAP) of the epididymal tail semen in camels with prolapsed prepuce. There were increases in progressive motility (PR), VCL, straight line velocity (VSL, μm/s), average path velocity (VAP, μm/s), BCF, and STR in the sperms of the group with phimosis, but the differences were non-statistically significant. Furthermore, the CASA parameters of the epididymal tail semen in the group with penile tumor showed an increase in PR (%), VCL, VSL, VAP, BCF, and STR (%) compared to those in the group with normal penis and prepuce. However, the CASA parameters of the epididymal body in the group with penile tumor showed a significant increase in vitality, total motility (TM), non-progressive motility (NP), mean angular degree (MAD), linearity (LIN, VSL/VCL), VSL, amplitude of lateral head displacement (ALH, μm), VAP, BCF, and STR compared to those in the group with normal penis and prepuce. There were no discernible differences in testosterone concentrations among the groups. There was a negative correlation (*p* < 0.05, *r* = 0.411–0.459) between testosterone concentration and CASA parameters of the epididymal tail semen in camels with penile and preputial pathological conditions. In comparison, there were no discernible differences in correlation (*p* > 0.5, *r* = 0.074–0.360) between testosterone concentration and CASA parameters of the epididymal body and head semen in camels with penile and preputial pathology. In conclusion, the semen quality of male dromedary camels could be affected by the penile and preputial pathological problems, while the testosterone concentration was not affected.

## Introduction

Dromedary camels account for 95% of the camel population globally, and they are predominantly found in Africa, especially the Middle East and Asia (1). A bulk of approximately 39,295,752 camels are located in African nations, with 7,425,979 in Somalia, 9,401,892 in Chad, 4,940,961 in Sudan, and 99,610 in Egypt, according to FAOSTAT (2). Dromedary camels are a traditional resource for human populations in desert areas, essential for social and economic life. Husbandry provides revenue from meat, milk, and secondary items (3).

Researchers and veterinarians have identified penile and preputial pathological issues such as balanoposthitis, penile trauma, preputial prolapse, phimosis, and penile tumors in male dromedary camels. Balanoposthitis is a common condition of inflammation that affects the penis and prepuce, causing a variety of disorders such as penile discomfort, pruritus, discharge, erythema, rash, and inconsolable crying (4). Penile trauma, at times called fracture of the penis, is the traumatic rupture of the tunica albuginea, the fibrous layer of the two cylinders (corpora cavernosa) that run down the penis (5). Preputial prolapse refers to the reversible turning of the foreskin inside out, resulting in one layer of the internal skin covering the outer layer (6). Phimosis is the inability to protrude the penis from the prepuce, a condition in which the penis opening narrows as a result of the foreskin retreating behind the glans, as well as the inability to retract the skin (foreskin or prepuce) surrounding the penis’ head (7, 8). Penile tumors are genital warts that are tiny growths or tumors seen on the genital region. Penile tumors are painless masses on the penis that might inhibit the foreskin from retracting and could lead to paraphimosis, the inability to retract the penis (9).

Clinicians and andrology researchers are much interested in computer-assisted semen analysis (CASA) systems and related algorithms (10). A crucial method for assessing the quality of semen in different species, CASA reduces human errors in manual analysis by providing objective assessments of sperm characteristics, which are critical for successful reproduction (11). Modern CASA systems have been designed to achieve high levels of intra- and inter-laboratory consistency and to determine objectively and quantitatively several aspects of the sperm structure and function (10, 12). Machine learning, multi-object tracking, localization, picture segmentation, and noise filtering techniques have been used to accomplish this goal (13). The cattle sector makes considerable use of CASA to assess the ejaculate quality in bulls (14). In addition to velocity measurements such as average path velocity (VAP), CASA assesses a number of motility parameters, such as total motility (TM) and progressive motility (PR) (15).

The mating process in camels plays a vital role in their herds, with male fertility often carried out by a single fertile male (16). Furthermore, camels exhibit a unique behavior during the mating process: ejaculation is frictional, copulation lasts 10–20 min, with three or four ejaculations, and mating frequently takes an hour (17). In addition, the traumas brought on by moving camels from one place to another might exacerbate penile and preputial pathologies. Studies on penile and preputial issues in male camels and their relationship to epididymal spermatozoa and future fertility are limited (18). The present study hypothesized that the penile and preputial pathology due to the aggressive nature of mating and injuries during transit could impair the semen quality. Hence, this study aimed at monitoring the penile and preputial pathological issues in male dromedary camels, as well as their influence on epididymal spermatozoa, using computer-assisted sperm analysis (CASA) and correlation analysis with testosterone concentrations.

## Materials and methods

### Animals

The animals were privately owned by farmers residing in Draw, Kom Ombo, Edfu, and Abu Sunbul in Aswan governorate, Aswan, Egypt, which is located at 24° 5′ 20′′ N latitude and 32° 53′ 59′′ E longitude on the eastern bank of the Nile River. It is located at approximately 900 km south of Egypt. The study was performed during the autumn of 2023. The animals were used in the study after obtaining an informed consent from all owners.

### Experimental design

A total of 60 sexually mature male dromedary camels, aged 8–10 years, were included in this study. The animals were examined for pathological abnormalities of the penis and prepuce, and they were subdivided into different groups (10 animals per group) based on the presence or absence of penile and/or preputial pathological problems: (i) animals with normal penis and prepuce, (ii) animals with balanoposthitis, (iii) animals with penile trauma, (iv) animals with preputial prolapse, (v) animals with phimosis, and (vi) animals with penile tumors, as presented in Table 1. A maintenance ration comprising 50% corn, 47% barley, 2% minerals, and 1% salt was supplied to camels at a rate of 3 kg/head/day. They were also provided with fresh water and Egyptian clover hay (*Trifolium alexandrinum*) on an as-needed basis. For the correct diagnosis of pathological conditions, the animals were held in the sternal recumbent position with ropes from the fore to hind limbs and a halter around their heads. After examining the heart rate, lungs, rumen, intestines, normal body temperature, respiration, feed intake, and regular movement, all animals were deemed clinically healthy (19).

**Table 1:** Grouping of male dromedary camels based on the penis and preputial pathological problems reported in this and previous studies.

Pathological problems (MP)	Identification	reference
Balanoposthitis	Various conditions affecting the penis and prepuce, such as penile pain, pruritus, discharge, erythema, rash, or inconsolable sobbing, are referred to as inflammation.	(4)
Penile trauma	A traumatic rupture of the tunica albuginea, the fibrous layer of the two cylinders, known as corpora cavernosa, which run along the penis.	(5)
Prolapsed prepuce	The reversible turning inside out of the foreskin is called prolapsed prepuce, denoting the appearance of one layer of the internal skin covering the outside layer	(6)
Phimosis	A condition where the penis opening narrows due to the foreskin retracting behind the glans, the inability to retract the skin (foreskin or prepuce) covering the head (glans) of the penis.	(8, 7)
Penile tumors	Genital warts are small growths, or tumors, on the genital area. Penile tumors present as painless lumps on the penis that can prevent the foreskin from retracting.	(9)

### Collection of epididymal spermatozoa

The animals were traced to a slaughterhouse (a local Aswan slaughterhouse), and a total of 120 testicles were collected after Islamic slaughter in 6 groups × 10 animals × 2 testes (right and left), preserved in normal saline solution (NSS), and transported to the laboratory within an hour. The testicles were examined after being cleaned with sterile normal saline (NSS). In order to gather a fluid rich in sperm, the cauda (tail), corpus (body), and caput (head) epididymides were separated, examined, cut lengthwise, and rinsed three to four times with Brackett and Oliphant medium in 60-mm Petri plates (Liverpool, Australia, Bacto Lab.). The plates were then placed on a warm stage at 37°C (20).

### Computer-assisted sperm analysis

**Figure 1:**
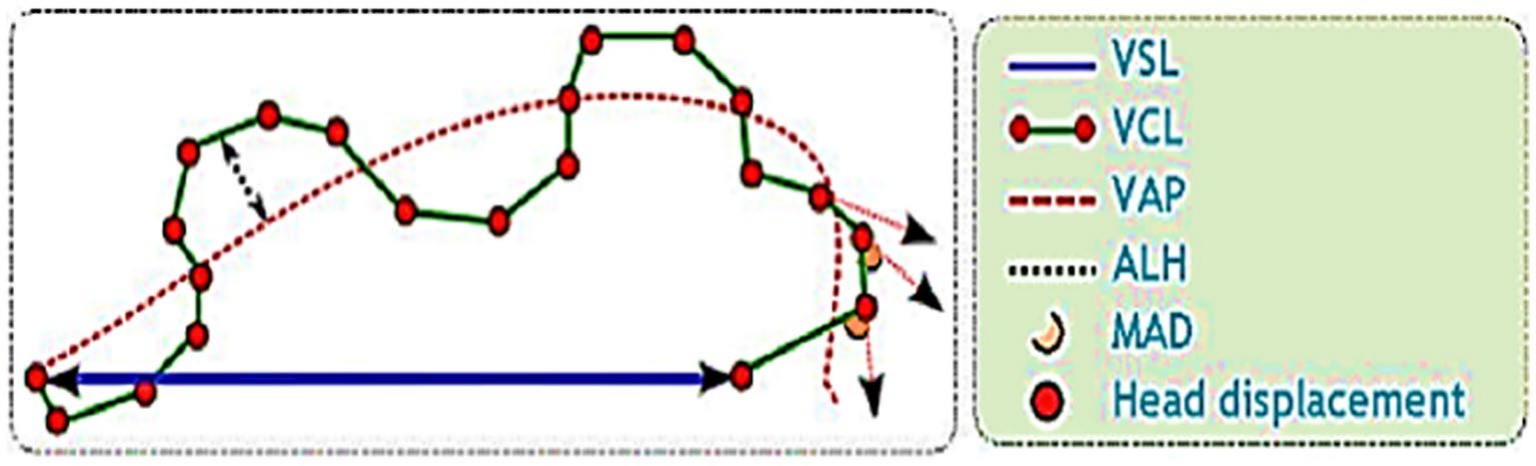
Diagram of sperm tracks and velocities with a spermolyzer (computer-assisted sperm analysis; CASA, version 12.2, IQM, Oslo, Norway) exhibits the method followed by the device to measure the parameters for monitoring epididymal spermatozoa in dromedary camels with penile and preputial pathologies and their correlation analysis with testosterone.

**Figure 2:**
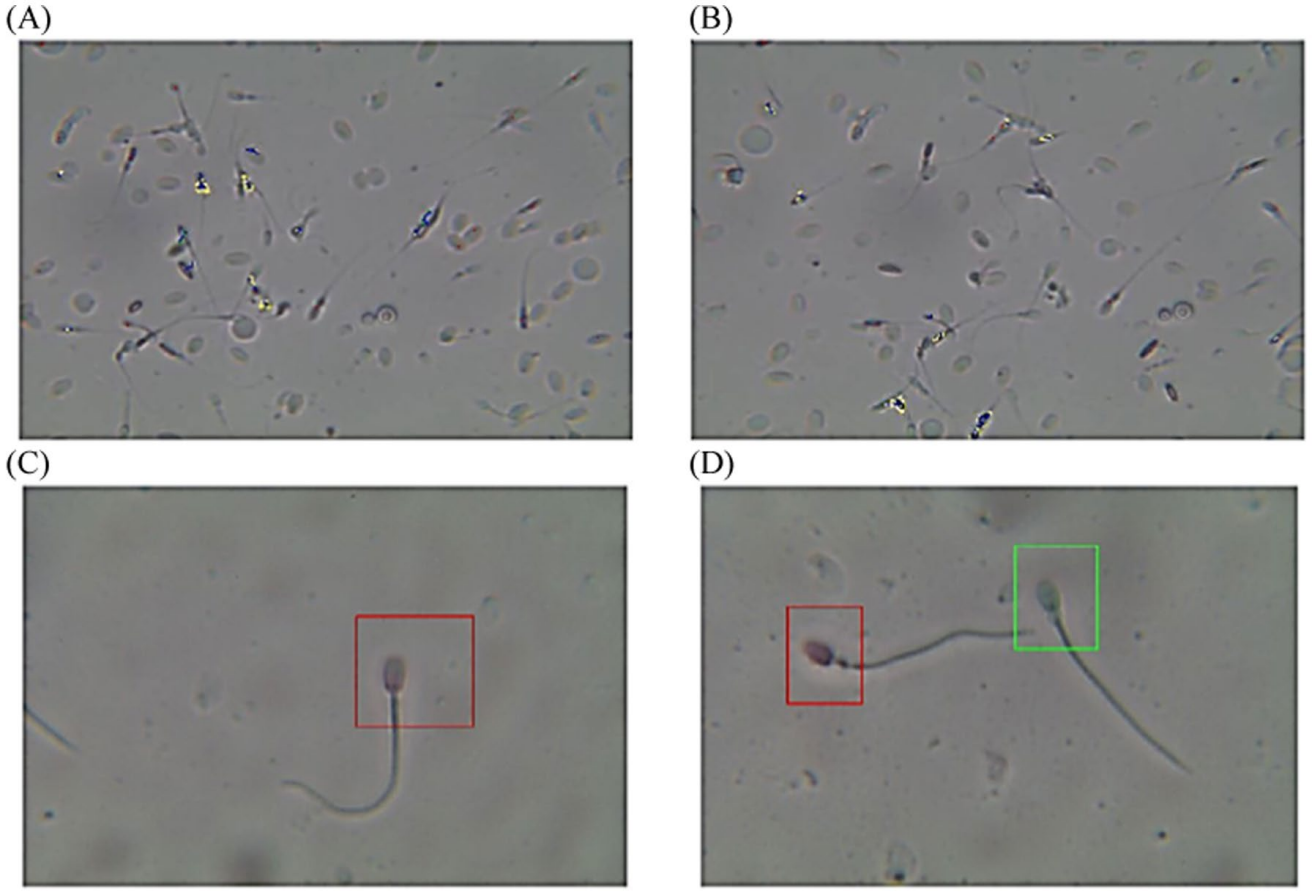
Diagram of sperm tracks and velocities with a spermolyzer (CASA) exhibits the method followed by the device to measure the parameters for monitoring balanoposthitis in dromedary camels. **(A,B)** 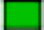 progressive motility, 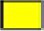 non progressive motility, 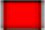 immotile; **(C,D)** 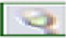 live, 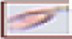 dead.

**Figure 3:**
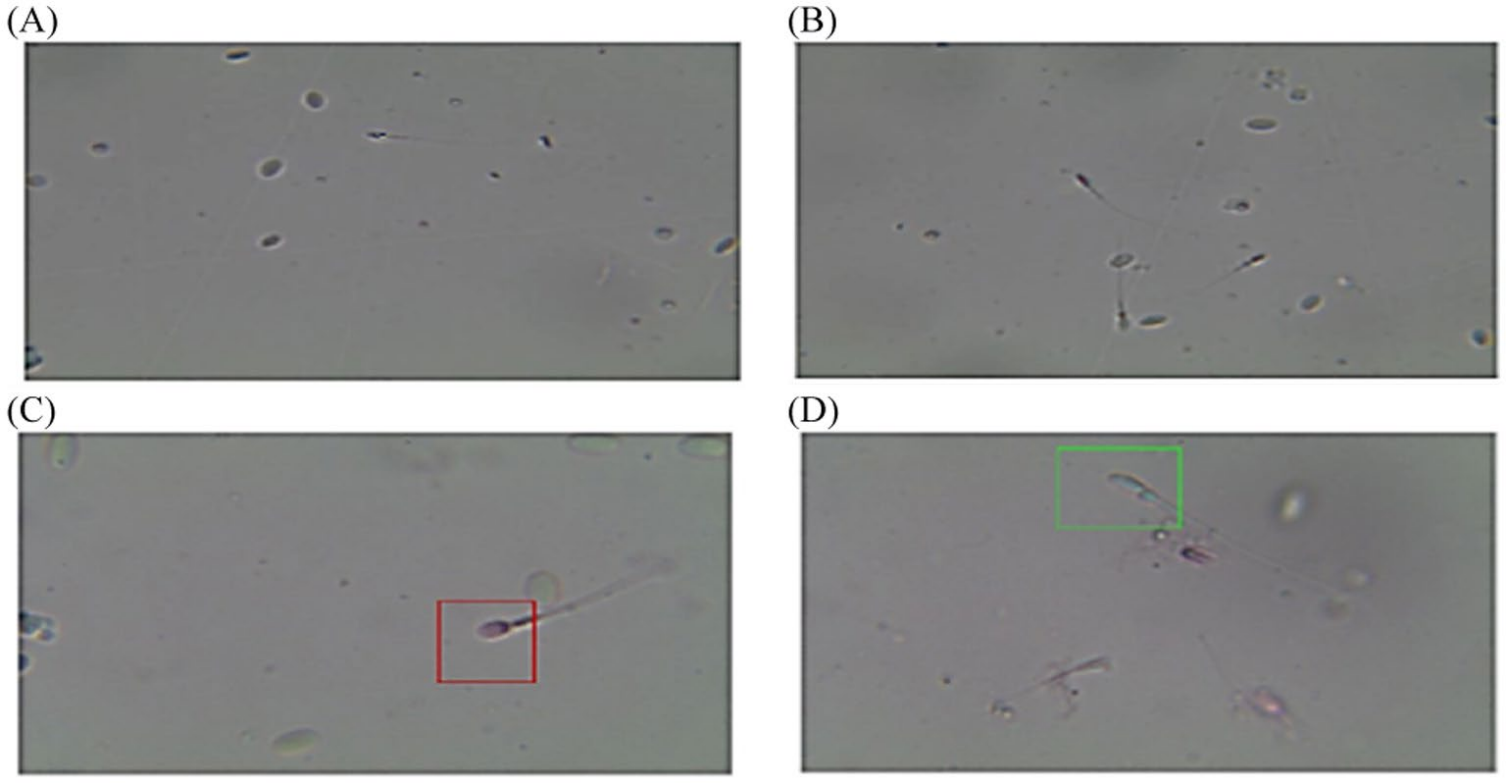
Diagram of sperm tracks and velocities with a spermolyzer (CASA) exhibits the method followed by the device to measure the parameters for monitoring penile trauma in dromedary camels. **(A,B)** 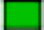 progressive motility, 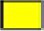 non progressive motility, 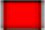 immotile; **(C,D)** 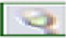 live, 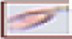 dead.

**Figure 4:**
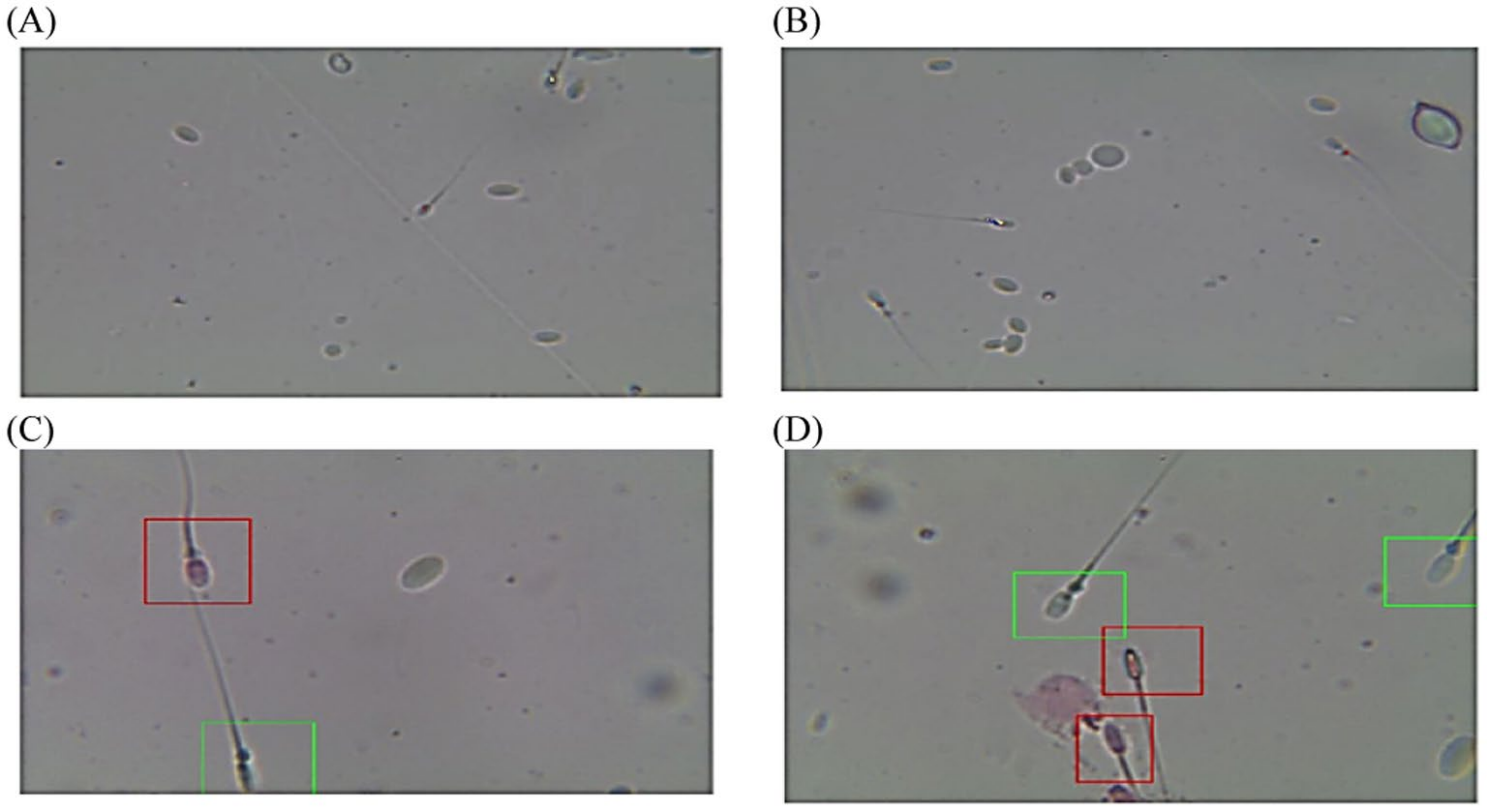
Diagram of sperm tracks and velocities with a spermolyzer (CASA) exhibits the method followed by the device to measure the parameters for monitoring prolapsed prepuce in dromedary camels. **(A,B)** 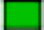 progressive motility, 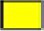 non progressive motility, 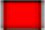 immotile; **(C,D)** 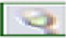 live, 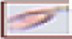 dead.

**Figure 5:**
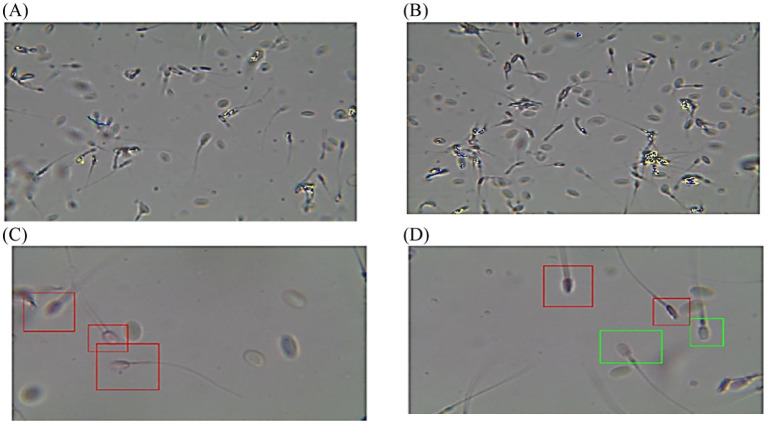
Diagram of sperm tracks and velocities with a spermolyzer (CASA) exhibits the method followed by the device to measure the parameters for monitoring phimosis in dromedary camels. **(A,B)** 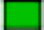 progressive motility, 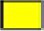 non progressive motility, 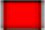 immotile; **(C,D)** 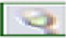 live, 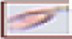 dead.

**Figure 6:**
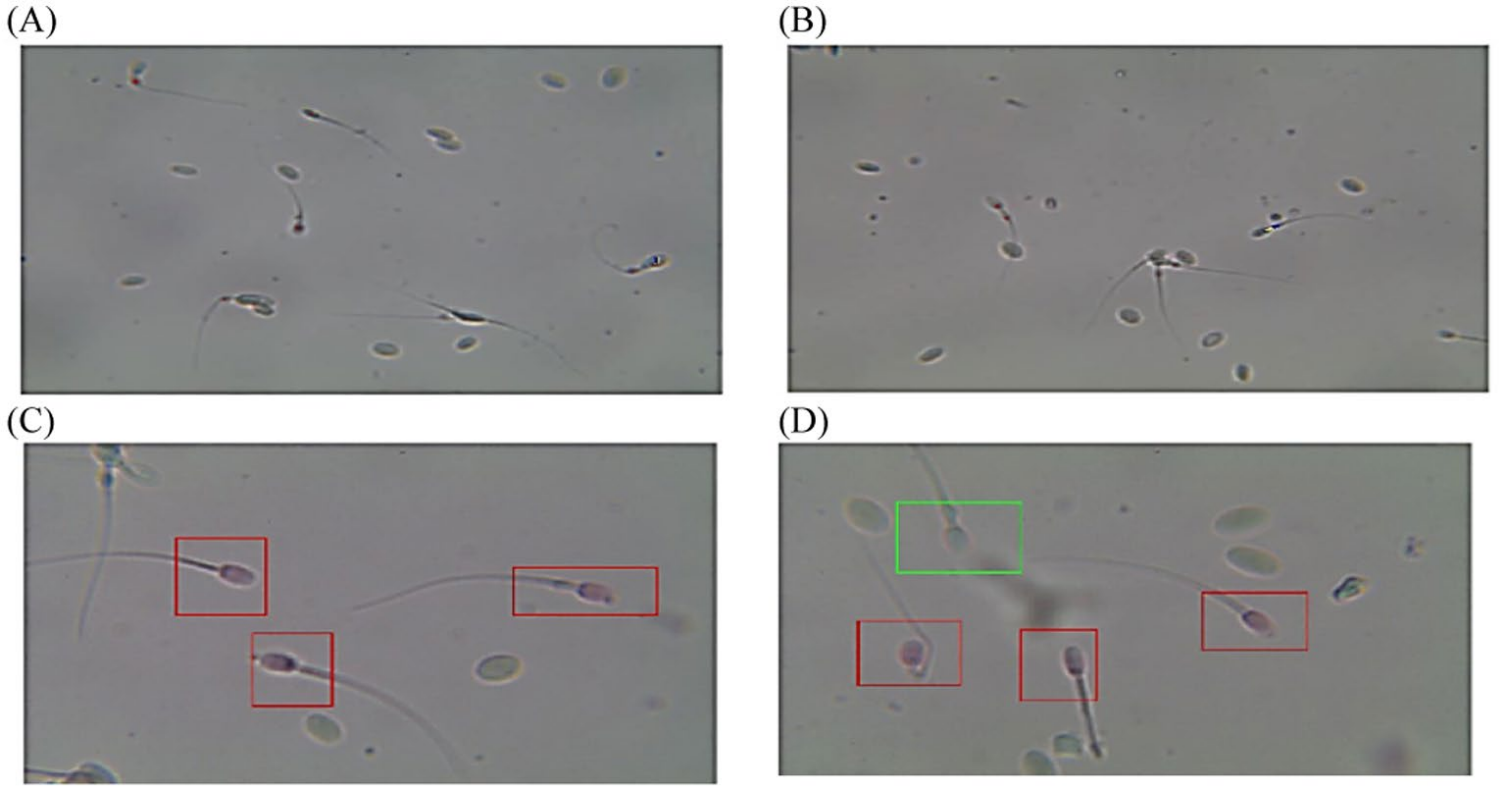
Diagram of sperm tracks and velocities with a spermolyzer (CASA) exhibits the method followed by the device to measure the parameters for monitoring penile tumors in dromedary camels. **(A,B)** 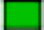 progressive motility, 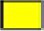 non progressive motility, 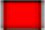 immotile; **(C,D)** 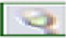 live, 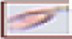 dead.

The post-thawed dromedary camel samples were evaluated for determining the characteristics of epididymal spermatozoa using a spermolyzer (computer-assisted sperm analysis; CASA, version 12.2, IQM, Oslo, Norway). The system followed the protocol defined by the World Health Organization for motility patterns and morphometric assessment of semen (Lab ID 202205891). Leja 4-chamber slides (IMV), with 20 μm deep, were warmed to 37°C and 30 μL of semen from each sample was held on each slide. Furthermore, fixed smears were stained with eosin and nigrosine stains and observed at 400x magnification to detect live spermatozoa (%). The spermatozoa that appeared with colored heads were considered to be dead, while those with colorless heads were considered to be live. In the analysis settings, spermatozoa were evaluated based on the following parameters: concentration (Conc., M/ml), total sperm count (TSC, M/ejaculate), vitality (%), total motility (TM, %), progressive motility (PR, %), non-progressive motility (NP, %), immotile (IM, %), curvilinear velocity (VCL, μm/s), mean angular degree (MAD), linearity VSL/VCL (LIN, %), straight line velocity (VSL, μm/s), amplitude of lateral head displacement (ALH, μm), wobble VAP/VCL (WOB, %), average path velocity (VAP, μm/s), eat-cross frequency (BCF, Hz), and straightness VSL/VAP (STR, %) (21, 22), as shown in Figure 1. Sperm tracks and velocities using a Spermolyzer (CASA), as well as the device’s method for measuring the parameters, were shown as follows: (Figure 2) monitors balanoposthitis group; (Figure 3) monitors penile injuries group; (Figure 4) monitors prolapsed prepuce group; (Figure 5) monitors phimosis group; and (Figure 6) monitors penile tumors group.

### Biochemical assays

A total of 60 blood samples (6 groups × 10 male camels × 1 time) were collected into plain and non-heparinized tubes by venipuncture on the day of slaughter in the slaughterhouse. Samples were centrifuged for 20 min at 3000× g* (gravity force), and the serum was collected and frozen at −20°C for testosterone assessment. The serum testosterone concentrations were measured using ELISA test kits (CA 94404; Biocheck Inc., Foster City, USA).

### Statistical analysis

The submitted data were statistically analyzed using SPSS for Windows 25 (SPSS, Chicago). The Kolmogorov–Smirnov test validated the normal distribution of the data. The data were analyzed using a one-way ANOVA, followed by Duncan’s multiple range test, according to the following general linear model:

Yij=μ+Ti+Aj+Eij

Where Yij represents the experimental observation, μ represents the general mean, Ti represents the groups (i = pathological problems and normal), and eij represents the experimental error.

## Results

### CASA results of the epididymal tail

The results of the epididymal semen analysis using CASA in normal camels and in those with penile and preputial pathology are presented in Table 2. The results of the parameters concentration (M/ml), vitality (%), MAD, LIN (%), ALH (μm), and WOB (%) showed a significant decrease in the balanoposthitis group compared to those in the normal penis and prepuce group. However, in the group with penile trauma, there was a significant decrease in the concentration (M/ml) and vitality (%) compared to those in the group with normal penis and prepuce. In the present study, there was a significant increase in the VCL (μm/s), BCF (Hz), and STR (%) in the group with preputial prolapse compared to those in the group with normal penis and prepuce. At the same time, the parameters did not indicate significant differences between the prolapsed prepuce and normal penis and prepuce groups. There was no statistical significance in the computer-assisted sperm analysis (CASA) parameters of the phimosis group when compared to those of the normal penis and prepuce group, other than a decrease in vitality (%). Furthermore, the CASA parameters showed an increase in PR (%), VCL (μm/s), VSL (μm/s), VAP (μm/s), BCF (Hz), and STR (%) in the penile tumor group compared to those in the normal penis and prepuce group (Table 2).

**Table 2:** Impact of penile and preputial pathology on the quality of the epididymal tail semen in dromedary camels.

Items	Normal	Balanoposthitis	Penile trauma	Prolapsed prepuce	Phimosis	Penile tumors	SEM	*P*-value
Conc. (M/ml)	6.48^a^	3.73^b^	3.80^b^	3.63^b^	6.48^a^	3.54^b^	0.40	0.017
TSC (M/ejaculate)	15.85^ns^	9.78^ns^	11.40^ns^	13.93^ns^	15.85^ns^	12.02^ns^	0.85	0.207
Vitality (%)	42.39^a^	19.00^b^	19.23^b^	49.75^a^	19.22^b^	45.13^a^	3.75	0.002
TM (%)	19.24^ab^	7.09^b^	20.51^ab^	25.48^ab^	19.24^ab^	33.02^b^	2.68	0.101
PR (%)	1.42^bc^	0.63^c^	0.64^b^	3.07^bc^	1.42^b^	4.20^a^	0.39	0.012
NP (%)	17.82^ab^	7.09^b^	20.20^ab^	22.41^ab^	17.82^ab^	28.82^a^	2.40	0.179
IM (%)	80.77^ab^	92.92^a^	79.50^ab^	74.53^ab^	80.77^ab^	66.98^b^	2.68	0.101
VCL (μm/s)	5.45^b^	2.76^b^	6.01^b^	11.55^a^	5.45^b^	13.72^a^	1.05	0.001
MAD (°)	29.52^ab^	10.16^c^	25.81^ab^	41.87^ab^	29.52^ab^	43.36^a^	3.19	0.006
LIN (%)	10.04^ab^	1.85^c^	7.57^bc^	13.19^ab^	10.04^ab^	14.98^a^	1.22	0.007
VSL (μm/s)	0.92^bc^	0.24^c^	0.80^bc^	1.56^ab^	0.92^bc^	1.93^a^	0.16	0.005
ALH (μm)	3.83^a^	0.92^b^	3.07^a^	5.03^a^	3.83^a^	5.00^a^	0.41	0.009
WOB (%)	30.10^a^	7.73^b^	24.80^ab^	39.60^a^	30.10^a^	42.67^a^	3.40	0.015
VAP (μm/s)	2.65^bc^	0.69^c^	2.42^bc^	4.57^ab^	2.65^bc^	5.52^a^	0.46	0.008
BCF (Hz)	2.21^bc^	1.04^c^	2.09^bc^	3.66^a^	2.21^bc^	3.81^a^	0.25	0.000
STR (%)	21.69^b^	15.40^b^	21.68^b^	31.15^a^	21.69^b^	33.82^a^	1.78	0.003

ns = significance at *p* > 0.05; a, b, c = Duncan test; Conc = concentration (M/ml); TSC = total sperm count (M/ejaculate); Vitality (%); TM (%) = total motility; PR (%) = progressive motility; NP (%) = non-progressive; IM (%) = immotile; VCL (μm/s) = curvilinear velocity. MAD (°) = mean angular degree; LIN (%) = linearity (VSL/VCL); VSL (μm/s) = straight line velocity; ALH (μm) = amplitude of lateral head displacement; WOB (%) = wobble (VAP/VCL); VAP (μm/s) = average path velocity; BCF (Hz) = eat-cross frequency; STR (%) = straightness (VSL/VAP).

### CASA results of the epididymal body

The CASA results of the epididymal body demonstrated the impact of penile and preputial pathology on the quality of the semen in dromedary camels, which are presented in Table 3. There was no statistical significance among the groups with regard to the computer-assisted sperm analysis (CASA) parameters concentration (M/ml), TSC (M/ejaculate), PR (%), and VCL (μm/s). The results of the epididymal body exhibited a significant increase in vitality (%), TM (%), NP (%), MAD (°), LIN (%), VSL (μm/s), ALH (μm), VAP (μm/s), BCF (Hz), and STR (%) in the penile tumor group compared to those in other groups. At the same time, the CASA parameters showed a decrease in IM (%; *p* < 0.051) in the penile tumor group compared to that in other groups (Table 3).

**Table 3:** Impact of penile and preputial pathology on the quality of the epididymal body semen in dromedary camels.

Items	Normal	Balanoposthitis	Penile trauma	Prolapsed prepuce	Phimosis	Penile tumors	SEM	*p*-value
Conc. (M/ml)	1.86^ns^	1.86^ns^	1.55^ns^	2.15^ns^	1.18^ns^	1.80^ns^	0.17	0.706
TSC (M/ejaculate)	4.04^ns^	4.04^ns^	2.78^ns^	4.47^ns^	5.07^ns^	3.90^ns^	0.41	0.787
Vitality (%)	16.84^b^	16.84^b^	20.84^b^	16.75^b^	18.75^b^	27.33^a^	1.03	0.001
TM (%)	6.90^b^	6.90^b^	6.85^b^	6.79^b^	6.76^b^	17.66^a^	1.24	0.024
PR (%)	1.07^ns^	1.08^ns^	0.78^ns^	1.14^ns^	0.98^ns^	1.12^ns^	0.08	0.853
NP (%)	5.83^b^	5.83^b^	6.08^b^	5.65^b^	5.78^b^	14.76^a^	0.99	0.013
IM (%)	93.10^a^	93.10^a^	93.15^a^	93.21^a^	93.24^a^	79.16^b^	1.69	0.051
VCL (μm/s)	1.53^ns^	1.53^ns^	2.57^ns^	1.50^ns^	2.02^ns^	4.40^ns^	0.39	0.230
MAD (°)	6.30^b^	6.30^b^	12.90^b^	6.37^b^	9.66^b^	29.64^a^	2.26	0.001
LIN (%)	1.49^b^	1.49^b^	3.33^b^	1.76^b^	2.68^b^	11.43^a^	1.03	0.008
VSL (μm/s)	0.14^b^	0.14^b^	0.26^b^	0.16^b^	0.22^b^	1.31^a^	0.13	0.023
ALH (μm)	0.79^b^	0.79^b^	1.63^b^	0.88^b^	1.30^b^	3.77^a^	0.29	0.001
WOB (%)	6.65^c^	6.65^c^	10.97^ab^	6.68^c^	8.84^bc^	13.88^a^	0.78	0.005
VAP (μm/s)	0.66^b^	0.66^b^	0.90^b^	0.63^b^	0.75^b^	3.42^a^	0.30	0.016
BCF (Hz)	0.77^b^	0.77^b^	1.16^b^	0.72^b^	0.92^b^	2.89^a^	0.22	0.003
STR (%)	3.54^b^	3.54^b^	9.44^b^	4.30^b^	7.24^b^	27.29^a^	2.34	0.002

ns = significance at *p* > 0.05; a, b, c = Duncan test; Conc = concentration (M/ml); TSC = total sperm count (M/ejaculate); Vitality (%); TM (%) = total motility; PR (%) = progressive motility; NP (%) = non-progressive; IM (%) = immotile; VCL (μm/s) = curvilinear velocity. MAD (°) = mean angular degree; LIN (%) = linearity (VSL/VCL); VSL (μm/s) = straight line velocity; ALH (μm) = amplitude of lateral head displacement; WOB (%) = wobble (VAP/VCL); VAP (μm/s) = average path velocity; BCF (Hz) = eat-cross frequency; STR (%) = straightness (VSL/VAP).

### CASA results of the epididymal head

The CASA results of the epididymal head revealed the impact of penile and preputial pathology on the quality of the epididymal head semen in dromedary camels, which are presented in Table 4. Furthermore, there was no significant difference among the groups with regard to the CASA parameters concentration (M/ml), TSC (M/ejaculate), vitality (%), TM (%), PR (%), NP (%), IM (%), VCL (%) (μm/s), MAD (°), LIN (%), ALH (μm), BCF (Hz), and STR (%) on the quality of the epididymal head semen in dromedary camels. However, pathological conditions in the penile and preputial areas, such as balanoposthitis, trauma, prolapse, phimosis, and tumors, resulted in substantial increases in WOB and VAP (μm/s) compared to those in the normal penis and prepuce group.

**Table 4:** Impact of penile and preputial pathology on the quality of the epididymal head semen in dromedary camels.

Items	Normal	Balanoposthitis	Penile trauma	Prolapsed prepuce	Phimosis	Penile tumors	SEM	*p*-value
Conc. (M/ml)	2.63^ns^	1.46^ns^	1.01^ns^	1.69^ns^	1.35^ns^	1.95^ns^	0.24	0.493
TSC (M/ejaculate)	6.44^ns^	4.65^ns^	2.66^ns^	5.65^ns^	4.15^ns^	6.80^ns^	0.75	0.682
Vitality (%)	21.35^ns^	30.26^ns^	43.50^ns^	23.64^ns^	33.57^ns^	37.14^ns^	3.09	0.319
TM (%)	17.27^ns^	20.29^ns^	20.46^ns^	20.20^ns^	20.33^ns^	15.05^ns^	1.08	0.662
PR (%)	0.51^ns^	0.56^ns^	0.56^ns^	0.56^ns^	0.56^ns^	0.52^ns^	0.01	0.633
NP (%)	17.69^ns^	19.73^ns^	19.90^ns^	19.64^ns^	19.77^ns^	14.53^ns^	1.02	0.659
IM (%)	85.20^ns^	79.71^ns^	79.54^ns^	79.80^ns^	79.67^ns^	84.94^ns^	1.30	0.638
VCL (μm/s)	1.00^ns^	1.88^ns^	3.35^ns^	1.14^ns^	2.25^ns^	3.21^ns^	0.36	0.267
MAD (°)	10.47^ns^	14.38^ns^	15.80^ns^	13.68^ns^	14.74^ns^	12.60^ns^	0.79	0.510
LIN (%)	6.50^ns^	8.56^ns^	8.12^ns^	8.78^ns^	8.45^ns^	5.84^ns^	0.58	0.653
VSL (μm/s)	0.33^b^	0.51^ab^	0.63^a^	0.45^ab^	0.54^ab^	0.52^ab^	0.04	0.032
ALH (μm)	0.81^ns^	1.18^ns^	1.59^ns^	0.97^ns^	1.28^ns^	1.43^ns^	0.10	0.248
WOB (%)	8.49^c^	10.47^ab^	11.86^a^	9.78^ab^	10.82^ab^	10.57^ab^	0.42	0.032
VAP (μm/s)	0.43^c^	0.72^b^	1.16^a^	0.50^b^	0.83^b^	1.10^a^	0.11	0.025
BCF (Hz)	0.74^ns^	1.18^ns^	1.56^ns^	1.00^ns^	1.28^ns^	1.31^ns^	0.10	0.279
STR (%)	17.54^ns^	20.75^ns^	22.41^ns^	19.93^ns^	21.17^ns^	17.34^ns^	0.96	0.651

ns = significance at *p* > 0.05; a, b = Duncan test; Conc = concentration (M/ml); TSC = total sperm count (M/ejaculate); Vitality (%); TM (%) = total motility; PR (%) = progressive motility; NP (%) = non progressive; IM (%) = immotile; VCL (μm/s) = curvilinear velocity. MAD (°) = mean angular degree; LIN (%) = linearity (VSL/VCL); VSL (μm/s) = straight line velocity; ALH (μm) = amplitude of lateral head displacement; WOB (%) = wobble (VAP/VCL); VAP (μm/s) = average path velocity; BCF (Hz) = eat-cross frequency; STR (%) = straightness (VSL/VAP).

### Testosterone concentrations

The results of testosterone concentrations under the pathological conditions in the penile and preputial areas in dromedary camels are presented in Table 5. Additionally, there were no discernible differences in testosterone concentrations among the groups in the present study.

**Table 5:** Testosterone concentrations under penile and preputial pathological conditions in dromedary camels.

Items	Normal	Balanoposthitis	Penile trauma	Prolapsed prepuce	Phimosis	Penile tumors	SEM	*p*-value
Testosterone	3.73^ns^	3.84^ns^	3.86^ns^	3.79^ns^	3.76^ns^	3.78^ns^	0.07	0.994

ns = significance at *p* > 0.05.

### Correlation (r) analysis

The results of the correlation analysis between testosterone concentrations and the CASA parameters of the epididymal semen (tail, body, and head) under the penile and preputial pathological conditions in dromedary camels are presented in Table 6. There was a negative correlation (*p* < 0.05, *r* = 0.411–0.459) effect among the testosterone levels and CASA parameters of the epididymal tail including TM (%; *p* < 0.050), NP (%; *p* < 0.051), IM (%; *p* < 0.041), VCL (μm/s; *p* < 0.042), MAD (*p* < 0.050), LIN (%; *p* < 0.050), VSL (μm/s; *p* < 0.049), ALH (μm; *p* < 0.041), WOB (%; *p* < 0.039), VAP (μm/s; *p* < 0.039), and BCF (Hz; *p* < 0.048), with *R*-values 0.459, 0.439, 0.459, 0.411, 0.416, 0.456, 0.448, 0.421, 0.418, 0.415, and 0.451, respectively, in the penile and preputial groups in dromedary camels. However, there were no discernible differences in the correlation (*p* > 0.5, *r* = 0.074–0.360) effect among the testosterone levels and CASA parameters of the epididymal body and head in the penile and preputial groups in dromedary camels.

**Table 6:** Correlation analysis between testosterone concentrations and CASA parameters of the epididymal semen (tail, body, and head) in dromedary camels.

Testosterone concentrations						
Items	Epidermal tail		Epidermal body		Epidermal head	
	*R*	*p-*value	*R*	*p-*value	*R*	*p-*value
Conc. (M/ml) =	0.001^ns^	<0.996	0.162^ns^	<0.508	0.161^ns^	<0.523
TSC (M/ejaculate)	0.029^ns^	<0.908	0.360^ns^	<0.130	0.128^ns^	<0.613
Vitality (%)	0.271^ns^	<0.278	0.294^ns^	<0.221	0.097^ns^	<0.721
TM (%)	0.459*	<0.050	0.207^ns^	<0.396	0.125^ns^	<0.622
PR (%)	0.370^ns^	<0.131	0.223^ns^	<0.358	0.152^ns^	<0.548
NP (%)	0.439*	<0.051	0.204^ns^	<0.403	0.112^ns^	<0.658
IM (%)	0.459*	<0.041	0.239^ns^	<0.324	0.143^ns^	<0.571
VCL (μm/s)	0.411*	<0.042	0.153^ns^	<0.533	0.066^ns^	<0.794
MAD (°)	0.416*	<0.050	0.221^ns^	<0.364	0.161^ns^	<0.524
LIN (%)	0.456*	<0.050	0.210^ns^	<0.388	0.124^ns^	<0.625
VSL (μm/s)	0.448*	<0.049	0.183^ns^	<0.454	0.149^ns^	<0.554
ALH (μm)	0.421*	<0.041	0.243^ns^	<0.317	0.106^ns^	<0.677
WOB (%)	0.418*	<0.039	0.271^ns^	<0.262	0.147^ns^	<0.562
VAP (μm/s)	0.415*	<0.039	0.177^ns^	<0.469	0.074^ns^	<0.769
BCF (Hz)	0.451*	<0.048	0.200^ns^	<0.411	0.133^ns^	<0.598
STR (%)	0.367*	<0.135	0.224	<0.357	0.143^ns^	<0.571

ns = significance at *p* > 0.05; * = significance at p < 0.05; Conc = concentration (M/ml); TSC = total sperm count (M/ejaculate); Vitality (%); TM (%) = total motility; PR (%) = progressive motility; NP (%) = non-progressive; IM (%) = immotile; VCL (μm/s) = curvilinear velocity. MAD (°) = mean angular degree; LIN (%) = linearity (VSL/VCL); VSL (μm/s) = straight line velocity; ALH (μm) = amplitude of lateral head displacement; WOB (%) = wobble (VAP/VCL); VAP (μm/s) = average path velocity; BCF (Hz) = eat-cross frequency; STR (%) = straightness (VSL/VAP).

## Discussion

The current study effectively sheds light on clear clinical understanding of penile and preputial pathological issues in male dromedary camels, including balanoposthitis, penile trauma, prolapsed prepuce, phimosis, and penile tumors. Computer-assisted sperm analysis (CASA) was used to investigate the semen parameters in penile and preputial pathology in male dromedary camels. Furthermore, the association of reproductive organs with semen characteristics enabled the semen quality prediction and the likelihood of future benefits. Limited research has been conducted on penile and preputial pathology in male camels, as well as their link to epididymal spermatozoa and future fertility (18).

The results of the concentration (M/ml), vitality (%), MAD, LIN (%), ALH (μm), and WOB (%) showed a significant decrease in camels with balanoposthitis compared to those with normal penis and prepuce as demonstrated by CASA results of the epididymal tail. Vasodilation, edema, cytotoxicity, and the facilitation of cytokine-dependent processes that may result in tissue destruction are examples of pro-inflammatory effects in the male genitalia (23). Therefore, pathological injuries may have caused a malfunction in the cells, leading to their functional impairment (24, 25).

The CASA parameters of the semen obtained from the epididymal body showed no significant difference among the groups with regard to concentration (M/ml), TSC (M/ejaculate), PR (%), and VCL (μm/s). The membranes connecting to the tail and the mitochondrial axonemal system, which govern motility, may be more susceptible to damage than the plasma membranes that surround the body and head (26). There is plenty of evidence that the plasma membrane varies among the various areas of spermatozoa (27). Spermatogenic failure, exhibited as azoospermia, is commonly associated with reduced Leydig cell activity (28). Such impaired functioning is mostly apparent due to histological and hormonal changes in the testis compartment (29).

There were no discernible differences in testosterone concentrations among the groups in the current study. Also, serum testosterone concentration and the prevalence of phimosis in impotent male camels were not found to be related. This could be attributed to the function of Leydig cells, which are responsible for testosterone hormone synthesis, not being altered due to the preputial or penile pathological problems. Additionally, similar results have been reported in male camels with pathologic lesions on their genital organs that caused phimosis (30).

In the present study, there was a significant increase in Conc. (M/ml), VCL (μm/s), BCF (Hz), and STR (%) in the prolapsed prepuce group compared to those in the normal penis and prepuce group. A variety of diseases, including orchitis and cryptorchidism, can cause spermatogenesis to be halted (31). The most common histological findings on testicular biopsy in Impotentia generandi camels were testicular degeneration, Sertoli cell-only syndrome, and halted spermatogenesis (32).

Severe trauma may cause Sertoli cell-only syndrome (33). However, this study found that CASA parameters of semen from the epididymal body of camels with penis and preputial pathological conditions, such as balanoposthitis, trauma, prolapse, phimosis, and tumors, resulted in substantial increases in WOB and VAP (μm/s) compared to those in the normal penis and prepuce group. Additionally, the Impotentia generandi camels showed low percentages of sperm motility and viability and a high rate of teratospermia (18). Numerous Impotentia generandi camels were found to be either oligo- or azoospermic when their semen was analyzed (34).

There was a negative correlation between testosterone concentration and the epididymal tail CASA parameters in camels with penile and preputial pathology. This could be attributed to the pathological problems of the penis and prepuce that have a negative effect on the spermatozoal activity and viability. On the other hand, Kumbhar et al. (35) found a positive correlation between the testicular length, mass activity, live sperm count, and early motility, which could be attributed to the positive effects of testicular length in the process of spermatogenesis and in turn sperm cell concentration.

Our study revealed no discernible differences in the correlation (r) effect among testosterone concentration and CASA parameters of the epididymal body and head in dromedary camels with penile and preputial pathology. According to Abu et al. (36), sperm motility and testicular morphology exhibited an insignificant correlation. At the same time, testicular morphometric characteristics in West African dwarf rams were reported to be positively correlated with sperm storage and production (37, 38).

## Conclusion

In conclusion, the semen quality in male dromedary camels could be affected by the penile and preputial pathological problems, while testosterone concentration was not affected.

## Data Availability

The original contributions presented in the study are included in the article/supplementary material, further inquiries can be directed to the corresponding author.
